# Interstitial lung disease is a dominant feature in patients with circulating myositis-specific antibodies

**DOI:** 10.1186/s12890-021-01737-7

**Published:** 2021-11-14

**Authors:** Abhinav K. Misra, Nathan L. Wong, Terrance T. Healey, Edward V. Lally, Barry S. Shea

**Affiliations:** 1 Division of Pulmonary, Critical Care and Sleep Medicine, Rhode Island Hospital and Alpert Medical School of Brown University, 593 Eddy Street, POB 224, Providence, RI 02903 USA; 2 Department of Medicine, Rhode Island Hospital and Alpert Medical School of Brown University, Providence, RI USA; 3 Department of Diagnostic Imaging, Rhode Island Hospital and Alpert Medical School of Brown University, Providence, RI USA; 4 Division of Rheumatology, Rhode Island Hospital and Alpert Medical School of Brown University, Providence, RI USA

**Keywords:** Interstitial lung disease, Myositis-specific antibodies, Polymyositis, Dermatomyositis

## Abstract

**Background:**

Many patients with polymyositis (PM) or dermatomyositis (DM) have circulating myositis-specific antibodies (MSAs). Interstitial lung disease (ILD) is a common manifestation of PM/DM, and it can even precede the onset of characteristic muscle or skin manifestations. Furthermore, there appear to be some patients with ILD and circulating MSAs who do not develop muscle or skin disease even after prolonged follow-up. We sought to determine whether ILD is equally or more common than myositis or dermatitis at the time of initial detection of MSAs.

**Methods:**

We identified all patients found to have circulating MSAs at our institution over a 4-year period and assessed for the presence of lung, muscle, and skin disease at the time of initial detection of MSAs. Among those found to have ILD, we compared demographic and clinical features, chest CT scan findings, and outcomes between those with PM/DM-associated ILD and those with ILD but no muscle or skin disease.

**Results:**

A total of 3078 patients were tested for MSAs, and of these 40 were positive. Nine different MSAs were detected, with anti-histidyl tRNA synthetase (anti-Jo-1) being the most common (35% of MSAs). Among patients with positive MSAs, 86% were found to have ILD, compared to 39% and 28% with muscle and skin involvement, respectively (*p* < 0.001). Fifty percent of all MSA-positive patients had isolated ILD, with no evidence of muscle or skin disease. Those with isolated ILD were more likely to be older and have fibrotic changes on chest CT, less likely to receive immunomodulatory therapy, and had worse overall survival.

**Conclusions:**

In this study we found that individuals with circulating MSAs were more likely to have ILD than classic muscle or skin manifestations of PM/DM at the time of initial detection of MSAs. Our findings suggest that the presence of ILD should be considered a disease-defining manifestation in the presence of MSAs and incorporated into classification criteria for PM/DM.

## Background

Polymyositis (PM) and dermatomyositis (DM) are major subtypes of idiopathic inflammatory myopathies (IIMs) and are typically characterized by proximal skeletal muscle weakness, muscle inflammation, and in DM typical skin manifestations [1, 2]. Patients with IIMs frequently have circulating autoantibodies, some of which are specific for IIMs and are known as myositis-specific antibodies (MSAs). MSAs include anti-aminoacyl tRNA synthetase antibodies (anti-Jo-1, anti-PL-7, anti-PL-12, anti-OJ, anti-EJ) as well as antibodies directed towards signal recognition particle (anti-SRP), nuclear matrix protein 2 (anti-NXP-2), transcription intermediary factor 1γ (anti-TIF1γ), melanoma differentiation-associated gene 5 (ant-MDA5), small ubiquitin-like modifier-1 activating enzyme (anti-SAE), and the Mi-2 antigen [3–13]. These antibodies are not detected in healthy individuals or in other autoimmune conditions but are found in 50–75% of individuals with IIM [14–17]. Interstitial lung disease (ILD) is a common manifestation of PM/DM and may be present at time of diagnosis or even precede onset of muscle or skin involvement [18–20]. It is believed to be the most common extra-skeletal manifestation of the disease [21, 22]. Some patients with ILD and MSAs do not develop overt myositis or dermatitis, and these patients might be underrecognized due to absence of muscle and skin findings [6, 19, 23–26]. We hypothesized that in patients with MSAs, ILD is more common than either muscle or skin involvement characteristic of PM/DM. In this study, we determined the frequency of lung, muscle, and skin disease at the time of positive MSA testing in a tertiary referral center. We also compared the clinical features of individuals with positive MSAs and isolated ILD and those with more “typical” PM/DM-associated ILD.

## Methods

### Study subjects

We identified all individuals for whom serum myositis specific antibodies (MSAs) were measured at the Lifespan health system (Rhode Island Hospital, The Miriam Hospital, and Newport Hospital) in Rhode Island, USA over the 4-year period from April 1, 2015 to March 30, 2019. This was done by searching the common electronic medical record (EMR) for all instances where the following tests were obtained: anti-Jo-1 antibodies, Myositis AssessR Panel (Quest Diagnostics), Myositis Specific 11 Antibodies Panel (Quest Diagnostics), and Myomarker Panel 3 (RDL Reference Laboratory). These antibody panels included both MSAs and myositis-associated antibodies (MAAs), but only those with positive MSAs were included in our cohort. The antibodies considered MSAs were the anti-synthetase Abs (Jo-1, PL-7, PL-12, EJ, and OJ) and antibodies to Mi-2, SRP, TIF1γ, NXP-2 and MDA-5. Individuals for whom clinical data were absent were excluded. This study was approved by the Lifespan Institutional Review Board.

### Clinical data collection

For all study subjects, the following information was collected, when available: age, sex, race, ethnicity, date of first positive MSA, results of additional autoimmune serologies, serum creatine kinase (CK) and aldolase levels, pulmonary function test (PFT) results, chest x-ray (CXR) or chest computed tomography (CT) results, thigh magnetic resonance imaging (MRI) results, electromyography (EMG) results, muscle biopsy findings, skin biopsy findings, and clinical documentation of symptoms or physical exam findings of lung, muscle, or skin disease (e.g. dyspnea, cough, lung rales, proximal muscle weakness, skin rash).

### Determination of organ involvement

Study subjects were assessed for the evidence of lung, muscle, or skin disease within 3 months of the date of the first positive MSA. Individuals were deemed to have ILD if they had characteristic findings of ILD on CXR or chest CT. Muscle involvement was identified by (1) serum CK or aldolase levels greater than 2 times of upper limit of normal or (2) symptoms or exam findings of muscle weakness associated with milder elevations of serum muscle enzymes or characteristic MRI, EMG, or muscle biopsy findings. Skin involvement was determined based on office notes and/or skin biopsy findings describing dermatitis characteristic of DM.

### ILD assessments

Study subjects identified to have ILD at the time of positive MSA were classified as having either acute/fulminant or chronic disease. Those with symptoms of < 3 months duration and/or requiring hospitalization for respiratory failure were determined to have acute/fulminant disease; all others were considered to have chronic disease. When chest CT scans were available, they were reviewed by an expert chest radiologist (T.T.H.) who was blinded to the clinical information, and presence and extent of ground glass opacities, reticular opacities, consolidation, traction bronchiectasis/bronchiolectasis, and honeycombing [2:23] were assessed with a semiquantitative scoring system (0 = none, 1 = mild, 2 = moderate, 3 = severe).

### Treatment and outcomes

The records of ILD subjects were reviewed to determine treatments given and outcomes following the date of ILD onset. Treatments were classified as corticosteroids, immunomodulators (azathioprine, mycophenolate, methotrexate, rituximab, or intravenous immunoglobulin), or antifibrotics (pirfenidone or nintedanib). Where available, subjects were classified as having either stability/improvement or progression of ILD. Progression was defined as either worsening parenchyma disease as seen on subsequent chest CT scans, a relative decline of FVC of ≥ 10% from baseline, death, or lung transplantation.

### Statistical analyses

Statistical analyses were performed with GraphPad Prism Version 8 software. Except where indicated, comparisons of continuous variables were performed using student’s t-tests or Mann–Whitney tests for parametric and nonparametric data, respectively. Chi-squared and Fischer exact tests were used for categorical data. Log-rank tests were used for Kaplan–Meier analyses of survival and progression-free survival.

## Results

**Fig. 1:**
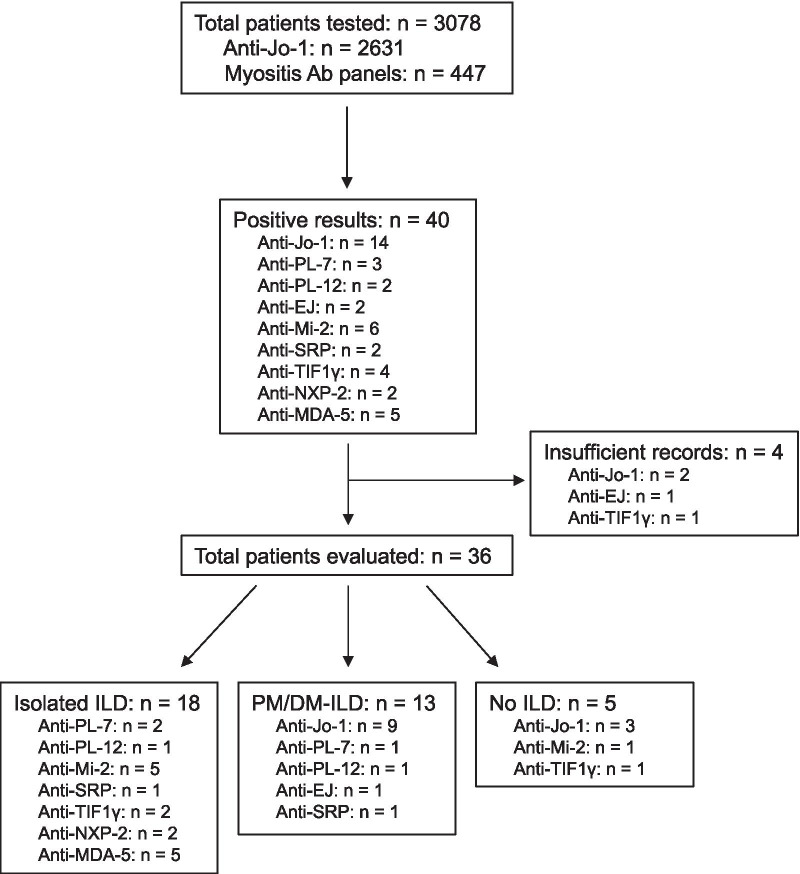
Schema for identification of study cohort

A total of 3078 serological tests for MSAs were obtained over the 4-year period from April 1, 2015 to March 30, 2019. Of these, 2631 tests (85.5%) were for anti-Jo-1 only and 447 (14.5%) were myositis antibody panels. A total of 40 different individuals were found to have positive MSAs, representing 1.3% of all tests sent. Nine different MSAs were detected; anti-Jo-1 was the most common but still made up only 35% (14/40) of MSAs detected (Fig. 1). There were insufficient records available for review for 4 of the individuals with positive MSAs, leaving a total of 36 subjects in our cohort. Subject demographics, smoking history, and serum CK values are shown in Table 1.

**Table 1:** Subject characteristics (N = 36)

Age—years (± SD)	58.9 (± 17.4)
*Sex*—*no. (%)*	
Female	19 (53)
Male	17 (47)
*Race*—*no. (%)*	
White	26 (72)
Black	4 (11)
Other/Unknown	6 (17)
Smoking history—no. (%)	16 (44)
Serum CK, IU/L—median (range)	108 (24–8015)

**Fig. 2:**
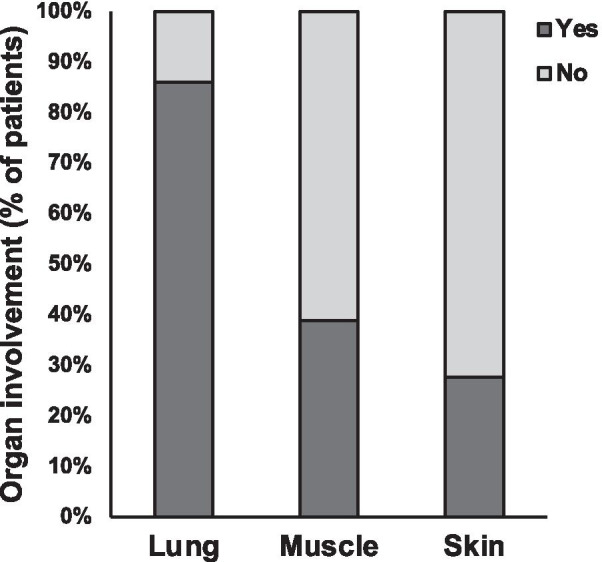
Frequency of lung, muscle, and skin involvement among the study population (n = 36) of individuals with positive circulating myositis-specific antibodies (MSAs). *p* < 0.0001 by Chi-squared test

The presence of ILD was significantly more common than muscle or skin disease at the time of first positive MSA (Fig. 2). ILD, muscle disease, and skin disease were present in 86% (31/36), 39% (14/36), and 28% (10/36) of the study subjects, respectively (*p* < 0.001). Only 50% (18/36) of the subjects would meet the ACR/EULAR criteria for probable or definite PM or DM [27]. The other 50% (18/36) of the subjects had isolated ILD and would not meet diagnostic criteria for PM or DM. Because full MSA panels may have been more likely to be obtained in individuals with unexplained ILD, we also analyzed the subset of patients who were positive for anti-Jo-1 antibodies, as testing for this antibody may be more likely to be obtained even in those patients with more clearly defined PM or DM. Among the anti-Jo-1 positive patients with sufficient data for analysis, ILD, muscle and skin disease were present in 75% (9/12), 83% (10/12) and 50% (6/12), respectively (*p* = 0.1824).

Basic demographic features were compared between the subjects with isolated ILD (n = 18) and those with clearly defined PM/DM-associated ILD (n = 13). Those with isolated ILD were significantly older and more likely to be White than those with PM/DM-ILD (Table 2), but the sex distribution was similar. There was a trend towards a difference in smoking status between the two groups, with the PM/DM-ILD subjects having numerically less smoking exposure, but this difference was not statistically significant.

**Table 2:** ILD subjects—demographics

	Isolated ILD (N = 18)	PM/DM-ILD (N = 13)	*p*
Age—years (± SD)	67.9 (± 14.1)	49.4 (± 16.9)	0.0023
*Sex*—*no. (%)*			0.41
Female	7 (39)	7 (54)	
Male	11 (61)	6 (46)	
*Race*—*no. (%)*			0.019
White	17 (94)	7 (54)	
Black	0 (0)	4 (31)	
Other/Unknown	1 (6)	2 (15)	
Smoking history—no. (%)	11 (61)	3 (23)	0.067

Clinical features, lung function, and CT scan findings were also compared between the subjects with isolated ILD and those with PM/DM-ILD (Table 3). Baseline lung function data were available for 13/18 (72%) of isolated ILD subjects and 11/13 (85%) of those with PM/DM-associated ILD. Severity of lung dysfunction, as determined by percent predicted for FVC and DLCO, was similar between the two groups. Chest CT scans were available to review for all 31 subjects with ILD. Those with isolated ILD showed more features suggestive of pulmonary fibrosis (reticulation and traction changes), while there were no appreciable differences in the amounts of ground glass opacities or consolidation. Interestingly, none of the ILD subjects’ chest CT scans showed honeycombing, and therefore none would be classified as showing a typical usual interstitial pneumonia (UIP) pattern of disease. Differences in the proportion of subjects who presented with acute/fulminant ILD were not statistically different between the two groups (33% of isolated ILD vs. 15% of PM/DM-ILD, *p* = 0.41). As expected, those with isolated ILD had lower median serum CK values than those with PM/DM-ILD (75 vs 265 IU/L, *p* = 0.0001). Those with isolated ILD were also less likely to have anti-synthetase antibodies compared to those with PM/DM-ILD (17% vs. 92%, *p* < 0.0001).

**Table 3:** ILD subjects—clinical features and treatment

	Isolated ILD (N = 18)	PM/DM-ILD (N = 13)	*p*
FVC % predicted—mean (± SD)	71.5 (± 21.1)	72.3 (± 22.5)	0.93
DLCO % predicted—mean (± SD)	49.7 (± 16.6)	64.4 (± 29.3)	0.15
*CT features*—*mean (*± *SD)*			
Ground glass	1.44 (± 0.86)	1.15 (± 0.80)	0.35
Consolidation	0.17 (± 0.51)	0.38 (± 0.77)	0.35
Reticulation	2.17 (± 0.79)	1.31 (± 0.63)	0.0029
Traction	1.89 (± 0.90)	0.92 (± 0.86)	0.0055
Fulminant ILD—no. (%)	6 (33)	2 (15)	0.41
Serum CK, IU/L—median (range)	75 (24–240)	265 (82–8015)	0.0001
Antisynthetase antibodies—no. (%)	3 (17)	12 (92)	< 0.0001
*Treatment—no (%)*	11 (65)	13 (100)	0.024
Prednisone	11 (65)	11 (85)	0.41
Immunomodulators	5 (29)	12 (92)	0.0008

**Fig. 3:**
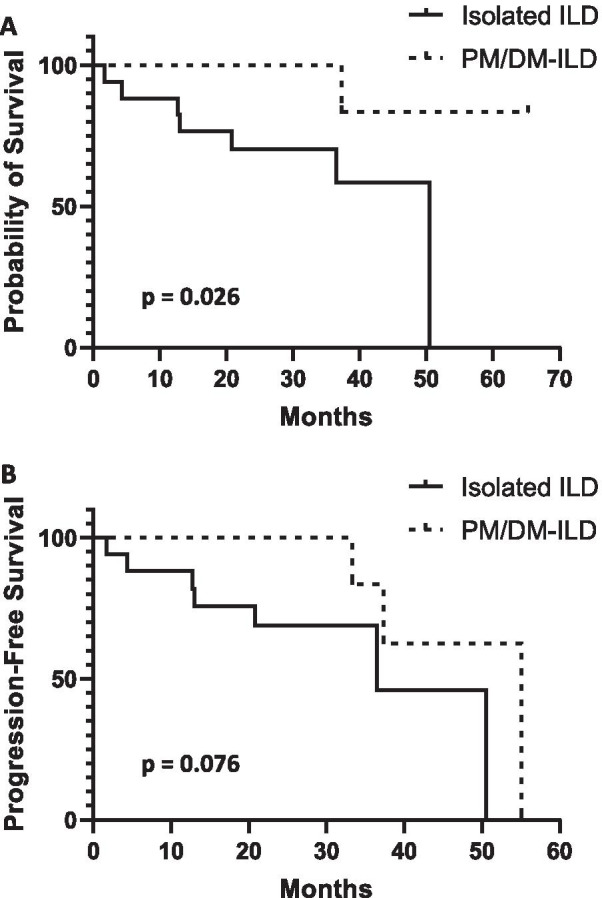
Kaplan–Meier curves of **A** survival and **B** progression-free survival in isolated ILD subjects vs. PM/DM-ILD subjects

Follow-up data were available for 17/18 (94%) of those with isolated ILD and for 13/13 (100%) of subjects with PM/DM-associated ILD. Those with PM/DM-ILD were significantly more likely than those with isolated ILD to receive any form of anti-inflammatory or immunosuppressive therapy (100% vs. 65%, *p* = 0.024), particularly immunomodulators (92% vs. 29%, *p* = 0.0008). In the isolated ILD group 7/17 (41.2%) subjects died during follow-up, compared to 1/13 (7.7%) of the PM/DM-ILD subjects. Kaplan–Meier survival analysis demonstrated significantly better survival in the PM/DM-ILD group (Fig. 3A). The cause of death could only be determined for 5 subjects, all in the isolated ILD group, and 4 of these were considered respiratory-related deaths. Among survivors, the presence or absence of disease progression was determined based on the last available chest CT scan or pulmonary function test. The mean duration of follow-up for determining disease progression among survivors was similar between the two groups (27.0 months for isolated ILD vs. 28.5 months for PM/DM-ILD, *p* = 0.79). When death or disease progression was used as a composite endpoint, the difference between the two groups was not statistically significant, but there was a trend towards improved progression-free survival in the PM/DM-ILD group (Fig. 3B). Survival was also compared between ILD patients with anti-synthetase (AS) antibodies and those with non-AS antibodies. While there was a trend towards poorer survival in the non-AS patients, the difference was not statistically significant (*p* = 0.096).

## Discussion

In this study, we assessed the frequency of lung, muscle, and skin disease in all individuals found to have circulating myositis-specific antibodies (MSAs) at a tertiary referral center over a 4-year period. Interestingly, we found that interstitial lung disease (ILD) was more common than classic muscle or skin manifestations of polymyositis (PM)/dermatomyositis (DM) at the time of positive MSA testing.

ILD is a frequent manifestation of PM/DM, and it has been reported to be more common in PM/DM patients with positive MSAs [21, 28]. There are also numerous descriptions in the literature of circumstances in which circulating MSAs are detected in individuals with ILD of unknown cause, in the absence of classic muscle or skin involvement typically associated with PM/DM [18–20]. In some of those cases, muscle and/or skin involvement develop after the onset of ILD, but in other cases such manifestations do not appear even with prolonged follow-up.

There has long been considerable interest in classifying individuals with ILD and features of autoimmunity who do not meet classification criteria for connective tissue diseases. The term interstitial pneumonia with autoimmune features (IPAF) has recently been proposed, along with a set of classification criteria, in an effort to better study and define this patient population [29]. The subjects in this study with positive MSAs and isolated ILD would likely meet criteria for IPAF. This is a potentially useful framework for approaching the diagnosis and management of such patients. However, grouping together all those individuals with ILD and features of autoimmunity as a single entity has the potential to minimize the importance of differences within that group of patients—e.g. those with isolated ILD and MSAs may behave more like PM/DM-associated ILD, while those with isolated ILD and positive anti-CCP antibodies may behave more like rheumatoid arthritis-associated ILD.

Anti-Jo-1 (anti-histidyl tRNA synthetase) was the most common MSA tested for and detected in this study, but it still made up only 35% on all MSAs in our cohort. The remaining 65% of MSAs were only detected through the use of more extended myositis antibody panels, despite the fact that these represented less than 15% of all MSA assays sent from our institution. These data are consistent with previous findings that although anti-Jo-1 is often the most common MSA found in PM/DM, it still makes up only 20–50% of all MSAs [17, 30]. Our findings suggest that more MSAs would have been detected had extended myositis antibody panels been sent more routinely. This is an important observation with direct clinical implications, as detecting a circulating MSA in cases where PM/DM is clinically suspected but not confirmed—or in individuals with ILD of unclear cause—may provide enough information to confirm a diagnosis without undergoing invasive procedures (e.g. lung biopsy). Moreover, in cases where isolated ILD presents as acute, fulminant respiratory failure of unclear etiology, detecting a positive MSA could allow for more timely initiation of life-saving treatments.

We observed some interesting demographic and clinical differences between those MSA-positive individuals with isolated ILD and those with PM/DM-associated ILD. Specifically, those with isolated ILD were more likely to be older and White, and their chest CT scans were more likely to show features suggestive of fibrosis (reticulation and traction changes). The reasons for these differences are unknown, but there are several potential explanations. It is possible that the disease biology of the ILD seen in MSA-positive individuals is different between those with and those without more classic PM/DM manifestations. Interestingly we found that those with PM/DM-ILD were more likely to have AS antibodies, while those with isolated ILD were more likely to have non-AS antibodies, and it has been well-described that different MSAs are associated with different disease phenotypes [31]. Patients with isolated ILD could be viewed as having a disease that is similar to idiopathic pulmonary fibrosis (IPF), although it is interesting that none of the ILD patients in this study had CT scan findings that would be considered typical for IPF [32]. However, it is also possible that those with isolated ILD were diagnosed later in the course of their disease, as patients with more clearly defined PM/DM would be more likely to have undergone aggressive screening for ILD and/or receive immunomodulatory therapy.

There were also differences in survival between those with isolated ILD and those with PM/DM-ILD, as the latter group had improved survival during follow-up. This difference in survival may be at least partially explained by differences in lung fibrosis, as the extent of fibrotic lung changes on chest CT has been correlated with poorer outcomes in a variety of ILDs [33–35]. However, it should also be noted that those with isolated ILD were less likely to receive anti-inflammatory treatment, particularly immunomodulators, and one could hypothesize that a more aggressive treatment approach in those with well-defined PM/DM led to improved survival. Those with isolated ILD were also more likely to have non-AS antibodies, while those with PM/DM-ILD were almost all AS antibody positive, and prior work has suggested that among MSA-positive ILD patients, the presence of anti-MDA-5 or other non-AS antibodies is associated with a poorer prognosis [15]. Other factors that may have contributed to the poorer survival in the isolated ILD group include older age and delay in diagnosis of ILD.

The role of MSAs in the etiology or pathogenesis of IIM is unknown. These autoantibodies do not appear to be involved with immune-complex formation nor are they known to mediate cellular cytotoxicity. Nonetheless, their association with IIM, particularly with relatively specific clinical sub-groups, is intriguing. MSAs may be genetically determined biomarkers that segregate with identifiable cohorts with the IIM phenotype (e.g. rapid ILD disease progression, specific dermatologic patterns, or malignancy). Alternatively, they could represent an epiphenomenon of the disease process. Regardless of their specific role(s) in IIM, our observations in this study emphasize the importance of serologic surveillance as part of the evaluation early in the disease process.

There are several limitations to our study. First, this was a retrospective analysis carried out at a single center, and our findings may not be generalizable to other institutions. The number of patients with skin manifestations of PM/DM may have been underestimated, as in the absence of a skin biopsy the presence or absence of skin disease could only be determined by review of clinical notes. Caution should also be taken in interpreting our survival analyses, as discussed above. There is also a possibility of sampling bias leading to an under-representation of individuals with more classic muscle or skin findings of PM/DM, who may have been less likely than patients with unexplained ILD to have extended myositis antibody panel testing done. Anti-Jo-1 testing is more likely to be routinely obtained in patients with well-defined PM or DM and therefore less likely to be influenced but sampling bias, and we did not detect significant differences in the frequencies of ILD, myositis, and dermatitis in the subset of patients with anti-Jo-1 antibodies. Nevertheless, ILD was still found in the majority of Jo-1-positive patients and at a similar rate as myositis, and it is possible that these differences in organ involvement reflect true differences in the disease process between those with AS and non-AS antibodies.

## Conclusions

**Fig. 4:**
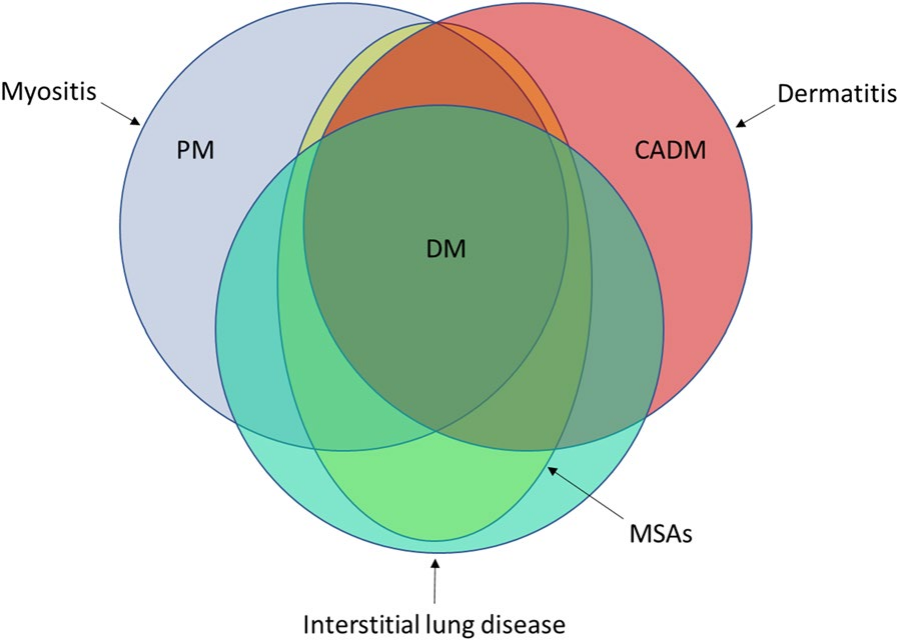
Non-proportional Venn diagram of lung, muscle and skin involvement in idiopathic inflammatory myopathies (IIM). The current view of IIM considers individuals with myositis, dermatitis, or both existing along a spectrum of illness, i.e. polymyositis (PM), dermatomyositis (DM), and clinically amyopathic dermatomyositis (CADM). Some of these individuals have circulating myositis-specific antibodies (MSAs), the presence of which can aid in the diagnosis. Incorporation of interstitial lung disease (ILD) into this disease paradigm would emphasize the true multisystem nature of this disease process and allow for more refined classification criteria

In summary, among all individuals with circulating MSAs identified at our institution over a 4-year period, we found ILD to be more common than classic muscle or skin manifestations of PM/DM at the time of MSA positivity. ILD should be considered a potential disease-defining manifestation when MSAs are detected, even in the absence of other organ involvement (Fig. 4), and consideration should be given to incorporating the presence of ILD into the classification criteria for PM/DM.

## Data Availability

All data generated or analyzed during this study are included in this published article. Requests to obtain the raw data used for this study should be directed to the corresponding author, Barry Shea, at bsshea@mgh.harvard.edu.

## Abbreviations

CXR: Chest X-ray
CT: Computed tomography
CK: Creatine kinase
DM: Dermatomyositis
EMG: Electromyography
IIM: Idiopathic inflammatory myopathy
IPF: Idiopathic pulmonary fibrosis
ILD: Interstitial lung disease
IPAF: Interstitial pneumonia with autoimmune features
MRI: Magnetic resonance imaging
MDA5: Melanoma differentiation-associated gene 5
MAA: Myositis associated antibody
MSA: Myositis specific antibody
NXP-2: Nuclear matrix protein 2
PM: Polymyositis
PFT: Pulmonary function test
SRP: Signal recognition particle
SAE: Small ubiquitin-like modifier-1 activating enzyme
TIF1γ: Transcription intermediary factor 1γ
